# Reliability and Validity of a Chinese Version of Urinary Tract Infection Symptom Assessment Questionnaire

**DOI:** 10.1590/S1677-5538.IBJU.2014.0046

**Published:** 2015

**Authors:** Shang-Jen Chang, Chia-Da Lin, Cheng-Hsing Hsieh, Ying-Buh Liu, I-Ni Chiang, Stephen Shei-Dei Yang

**Affiliations:** 1 Division of Urology, Taipei Tzu Chi Hospital, Buddhist Tzu Chi Medical Foundation, Taipei Taiwan, and Medical College of Buddhist Tzu Chi University, Hualien, Taiwan; Medical College of Buddhist Tzu Chi University, Hualien, Taiwan; 2 Department of Urology, National Taiwan University Hospital, Taipei, Taiwan

**Keywords:** Urinary Tract Infections, Lower Urinary Tract Symptoms, Questionnaires, Social Validity, Research, Validation Studies as Topic

## Abstract

**Objectives:**

Our study evaluates the reliability and validity of a Chinese version of the Urinary Tract Infection Symptom Assessment questionnaire (UTISA).

**Material and Methods:**

Our study enrolled women who were diagnosed with uncomplicated urinary tract infection (uUTI) at clinics. The Chinese version of UTISA was completed upon first visit to the clinic for uUTI and at 1-week follow-up. We enrolled 124 age-matched women without uUTI from the community as the control group. The UTISA consists of 14 items (seven symptom items and seven related to quality of life), with each item scoring 0 to 3. The internal consistency was assessed with Chronbach's alpha test. Factor analysis was used to classify symptoms into latent factors. The predictive validity was analyzed by using logistic regression and Receiver Operating Characteristic (ROC) curve analysis.

**Results:**

Mean total symptom scores of the UTISA in the 169 cases and 124 controls were 8.9±4.6 and 1.4±2.4, respectively (p<0.01). The alpha coefficient was 0.77, showing a homogeneous composition of symptoms. At a cut-off value of greater than 3, the UTISA symptom score had good predictive value for uUTI (sensitivity of 87.0%, and specificity of 93.1%). Factor analysis revealed two latent variables: 1) lower urinary tract symptoms and 2) physical symptoms. Among the seven items, we found that urinary frequency (OR=2.6), dysuria (OR=5.0), sense of incomplete emptying (OR=2.0), and hematuria (OR=7.6) were significant predictors for uUTI.

**Conclusions:**

The Chinese version of UTISA is reliable to predict uncomplicated UTI in women with an optimal cut-off point at >3.

## INTRODUCTION

It is estimated that one third of women experience at least one episode of urinary tract infection (UTI) by the age of 24 years old, and more than one half of all women experience at least one episode of UTI throughout their lifetime (1). Though untreated uncomplicated urinary tract infections (uUTI) rarely progress to life-threatening diseases (2), it leads to marked impairment of the quality of life in women (3, 4). Diagnosis of uUTI is established by symptoms and a positive urine culture. However, physicians usually treat uUTI based on self-reported symptoms and physical examination (5, 6). Symptoms of uUTI include burning or pain and also urinating, urinary frequency, urge to void, blood in the urine, and lower abdominal discomfort. However, there was no validated questionnaire scale to measure the severity of uUTI symptoms in patients until Clayson et al. (7) first developed the urinary tract symptoms assessment (UTISA) questionnaire in 2005. The questionnaire has been validated in English and showed good validity and responsiveness to change of uUTI severity. As no such validated scale exists in Chinese for measuring symptoms of UTI, we performed a case-control study to evaluate the reliability and validity of a Chinese version of the UTISA. We also used factor analysis to explore the latent factors underlying the symptoms of uUTI.

## MATERIALS AND METHODS

The UTISA is a self-administered questionnaire consisting of 14 items, with scores for each item ranging from 0 to 3 (Appendix-1). Among these items, seven were related to severity of symptoms and seven were related to quality of life. After approval of the original author, the UTISA was translated into Chinese by one urologist (SJ Chang) and one expert in English translation. The Chinese UTISA (Appendix 2) was also reviewed by three urologists from our institution for content validity and equivalence. Then, we performed a case-control study to validate the Chinese UTISA. From Jan 2012 to July 2013, we enrolled adult women with a diagnosis of uncomplicated UTI through clinical evaluation. The diagnostic criteria for uncomplicated UTI were symptomatic patients with bacteriuria >10^3^ cfu/mL according to international guidelines (8). Exclusion criteria were those with fever >38ºC, pregnancy, urolithiasis, genitourinary tract anomaly, under immuno-suppressive therapy, recent antibiotics use (within one month), chronic kidney disease under dialysis, and chronic urine retention under urethral catheterization. We recorded baseline characteristics including age, urine analysis, and urine culture. The questionnaire was completed at the clinic by the patient under the guidance of a study nurse. One week after antibiotics treatment, patient received urinalysis and completed follow up UTISA at the clinic. As for the control group, we enrolled 124 age-matched healthy adult women without pyuria, nitrite, or leukocyte esterase on urine analysis from the community. The exclusion criteria for the control group were women who had a urinary tract infection within the past month, history of urolithasis, or a neurogenic bladder.

Data was expressed as mean ± standard deviation and analyzed by commercial statistical software (SAS®, version 9.3, SAS Institute Inc., NC, USA). Demographic and voiding parameters were compared via an independent samples t-test (continuous demographic variables), χ^2^ test (nominal data), and Mann-Whitney U test (ordinal data). The values of missing items were given as the mean value of other completed items by that case. The reliability of the UTISA questionnaires completed by the cases was assessed using Chronbach's alpha test (internal consistency). Spearman rank correlation test was used to evaluate the correlation between symptoms and symptom related quality of life. Through the age-matched case control study, the predictive validity was analyzed by using logistic regression and Receiver Operating characteristic (ROC) curve analysis. Factor analysis was used to classify the seven symptom items into latent factors. The number of factors explored was determined by a screen plot and principal components analysis. We conducted factor analysis on the correlation matrix using the FACTOR procedure in SAS v.9.3. A varimax rotation was used. Squared multiple correlations were used as prior communality estimates. An item was assumed to load on a factor if the factor loading was at least 0.30 for that factor and less than 0.30 for all other factors.

## RESULTS

In total, there were 169 and 124 women (mean age; 44.6 vs. 44.0 years old, p=0.90), respectively, with and without uUTI enrolled for analysis. Table-1 lists the baseline characteristics and individual UTISA items of the cases and control group. The prevalence of each symptom (Q1 to Q7) in patients with uUTI were 88.2%, 76.3%, 75.1%, 76.3%, 64.5%, 36.1% and 49.7%, respectively. There were no significant differences in the ages between the uUTI patients and the control group. The mean total symptom scores of the UTISA in the cases and controls were 8.9±4.6 and 1.4±2.4, respectively (p<0.01). Each item of the UTISA symptom score in the cases was significantly higher than in the control group. As shown in the Table-2, strongly positive correlations between the severity of the symptom and the symptoms related QoL were observed.

**Table 1:** Comparisons of baseline characteristics, symptom scores between control and cases.

Parameters	Control (n=124)	Cases (n=169)	p-Value
Age (years)	44.6±12.3	44.0±13.7	0.90
Q1 (urinary frequency)	0.5±0.6	1.7±.9	<0.01
Q2 (urgency)	0.3±0.6	1.4±1.0	<0.01
Q3 (dysuria)	0.1±0.30	1.5±1.1	<0.01
Q4 (sense of incomplete emptying)	0.2±0.5	1.4±1.0	<0.01
Q5 (lower abdominal discomfort)	0.2±0.4	1.1±1.0	<0.01
Q6 (low back pain)	0.1±0.4	0.7±1.0	<0.01
Q7 (hematuria)	0.04±0.2	0.9±1.1	<0.01
Total Score	1.4±2.4	8.9±4.6	<0.01

**Table 2:** Correlations between severity of symptoms and related QoL.

QoL	Case (n=169)	Correlation coefficient with symptoms	p-value
Q8 (urinary frequency related QoL)	1.6±1.0	0.80	<0.01
Q9 (urgency related QoL)	1.3±1.0	0.87	<0.01
Q10 (dysuria related QoL)	1.4±1.0	0.89	<0.01
Q11 (sense of incomplete emptying related QoL)	1.4±1.0	0.86	<0.01
Q12 (lower abdominal discomfort related QoL)	1.1±1.0	0.85	<0.01
Q13 (low back pain related QoL)	0.7±.9	0.80	<0.01
Q14 (hematuria related QoL)	0.9±1.0	0.81	<0.01

### Reliability

The Chronbach's alpha coefficient was 0.77 for the seven symptom items in the UTISA, showing a homogeneous composition of symptoms in uUTI.

### Factor analysis

We used a factor analysis to explore the latent factors underlying the symptoms of uUTI. Through the factor analysis of the UTISA completed by the cases, we found two latent variables: Factor 1, lower urinary tract symptoms (Q1 urinary frequency, Q2 urgency, Q3 dysuria, Q4 sense of incomplete emptying, Q7 hematuria); Factor 2, physical symptoms (Q5 lower abdominal discomfort, Q6 low back pain). The distribution of the items and their respective factor loadings on the extracted factors were listed in the Table-3.

**Table 3:** The distribution of the items and their respective factor loadings on the extracted factors.

Parameter	Factor 1	Factor 2
Q1 (urinary frequency)	0.68	0.15
Q2 (urgency)	0.68	0.16
Q3 (dysuria)	0.62	0.15
Q4 (sense of incomplete emptying)	0.63	0.28
Q5 (lower abdominal discomfort)	0.28	0.59
Q6 (low back pain)	0.02	0.59
Q7 (hematuria)	0.32	0.05

### Receiver Operating Characteristic (ROC) curve analysis

Using the ROC curve analysis for the total UTISA symptom score, the optimal cut-off points of the UTISA symptom was >3 with a sensitivity of 87.0% and a specificity of 93.1%. The Area under the Curve (AUC) was 0.94.

Area under curve of each symptom is listed in Table-4. Q3 (Dysuria) and Q1 (urinary frequency) had highest AUC. Table-4 shows the results of the multivariate logistic regression. Among the seven symptom items in the UTI-SA, we found that Q1 (OR=2.6), Q3 (OR=5.0), Q4 (OR=2.0), and Q7 (OR=7.6) were significant predictors for uUTI.

**Table 4:** Area under curve and odds ratio of age and each symptom.

Parameter	Area Under Curve	Odds Ratio	95 Confidence Interval of OR
Age	0.51	1.01	0.98 to 1.04
Q1 (urinary frequency)	0.85	2.60	1.38 to 4.89
Q2 (urgency)	0.80	0.85	0.44 to 1.65
Q3 (dysuria)	0.86	5.00	2.24 to 11.21
Q4 (sense of incomplete emptying)	0.84	2.05	1.09 to 3.84
Q5 (lower abdominal discomfort)	0.78	1.15	0.55 to 2.38
Q6 (low back pain)	0.66	1.13	0.58 to 2.22
Q7 (hematuria)	0.74	7.61	2.23 to 26.0

### Responsiveness to change

**Figure 1:**
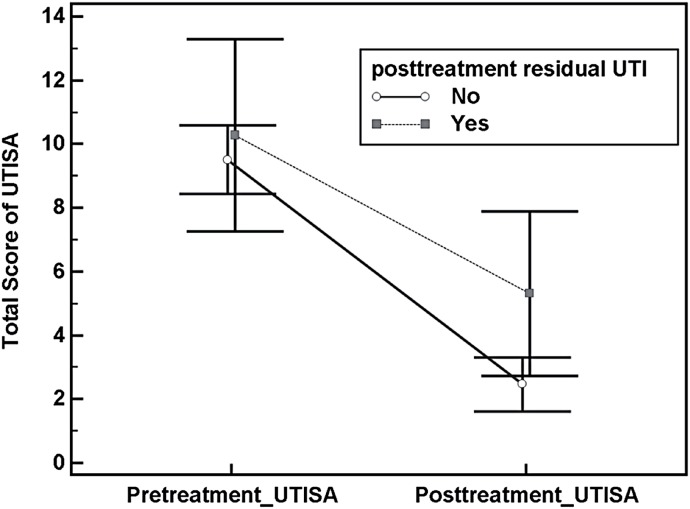
Comparison of changes in total score of UTISA in patient with and without residual UTI at 1 week follow-up.

After one week of antibiotics treatment, 71 patients had follow up data for UTISA. The symptom score of the severity of symptoms decreased significantly from 9.8±4.7 to 2.9±3.5 (p<0.01). Patients without pyuria, leukocyte esterase or nitrite on follow up urine analysis had significantly lower total score of UTISA than those with positive pyuria, leukocyte esterase or nitrite (p=0.03) (Figure-1).

## DISCUSSION

Our study showed that Chinese version of UTISA has good reliability and predictive validity. The Chinese UTISA questionnaire had high internal consistency with an alpha coefficient of 0.77 implying a homogeneous composition of symptoms in women with uUTI (9). The chosen optimal cut-off point for the total UTISA questionnaire symptom score was >3 with a sensitivity of 87.0% and specificity of 93.1%. As compared to the women without UTI, those with uUTI had higher scores in each item of the UTISA. Among the seven symptom items in the UTISA, we found that Q1 urinary frequency (OR=2.6), Q3 dysuria (OR=5.0), Q4 sense of incomplete emptying (OR=2.0), and Q7 hematuria (OR=7.6) were significant predictors for uUTI. Through the exploratory factor analysis of the symptom scores in UTISA questionnaire completed by the women with uUTI, we identified two latent factors (Factor 1: lower urinary tract symptoms; Factor 2: physical symptoms). Responsiveness to change, an ability of an instrument to measure a clinical change in a clinical state, of UTISA correlated well with the results of urine analysis. In the patients without uUTI on follow-up, the symptom score improved more significantly as compared to those with uUTI on follow up (Figure-1, p=0.03).

This is the first study using ROC curve analysis to determine the optimal cut-off point for total UTISA symptom score to differentiate patients with and without uUTI. The chosen cut-off point for total UTISA symptom score to predict women with uUTI was >3. The original study of UTISA did not enroll women without uUTI as control group and, therefore, did not evaluate the predictive validity of UTISA (7). The UTISA is also under validation in Korean, while predictive validity was not reported (10). Recently, Alidjanov et al. (11) developed a self-reporting questionnaire, acute cystitis symptom score, to assess the symptoms of urinary tract infections. They found that symptom score was significantly higher in patients with uUTI and they proposed an optimal threshold score at 6 points (sensitivity: 94% and specificity: 90%). However, the questionnaire is in the Russian and Uzbek language.

In the review by Bent et al. (12), four symptoms, i.e. dysuria, urinary frequency hematuria and back pain, increased the probability of uUTI in women. However, previous studies only asked if patients have the specific symptom and did not use a validated measurement scale to evaluate the symptom of uUTI in women. Our study used multivariate logistic regression analysis to determine risk of individual symptom on uUTI. We found that Q1 urinary frequency (OR=2.6), Q3 dysuria (OR=5.0), Q4 sense of incomplete emptying (OR=2.0), and Q7 hematuria (OR=7.6) are most significant predictors for uUTI in adult women. The area under the curve of ROC of each symptom was highest in dysuria, frequency and urgency. Although patients with hematuria had highest odds for having uUTI, the AUC of hematuria is relatively lower due to the lower prevalence of hematuria.

We did not measure the test-retest reliability because the symptoms associated with uUTI, such as urinary frequency and dysuria, would change very quickly with time. Our results showed that total UTISA score in patients with uUTI decreased significantly after antibiotics treatment showing a good responsiveness to change. As shown in the Figure-1, patients with persistent urinary tract infections had significantly higher total UTISA scores than those without uUTI on follow up. The results supported that UTISA can help us identify those with persistent uUTI after treatment. Through the validation of UTISA we could use the measurement scale to assess the response of women with uUTI to different regimen and duration of antibiotics.

In the current study, we used exploratory factor analysis to classify the 7 symptom items into two factors (Factor 1, lower urinary tract symptoms and Factor 2, physical symptoms). The original author also did factor analysis for UTISA and they classified symptom severity and symptom related QoL into four factors (urination regularity, problems with urination, pain associated with UTI and blood in urine). However, the reason why we did factor analysis is that we want to know the underlying latent factors that construct the symptoms of uUTI while not the latent factors that construct all the items in the questionnaire. Therefore, we did not analyze all symptom and QoL items all together with factor analysis.

There are several limitations in our study. The first major limitation is that the Chinese version of UTISA is a translated questionnaire. We did not increase the items and change measurement scale for construct and content validity. Other symptoms including vaginal discharge or irritation which were negative indicators of uUTI were not included (12). Second, we did not conduct informant interviews to learn whether the patients found any of the items too difficult to answer. Finally, the study design was an age-matched case-control study. As compared to cross-sectional study, a case-control study may eliminate the effect of age on the predicted probability of the UTISA.

## CONCLUSIONS

The Chinese version of UTISA is reliable with validity to predict uncomplicated UTI in women. We chose the cut-off point of the total UTISA symptom score at >3 to predict women with uncomplicated UTI. The composition of symptoms associated with uUTI in women is homogeneous and can be classified into two latent factors including lower urinary tract symptoms and physical symptoms.

## APPENDIX 1

**1A**

**1B**
